# Carboxypeptidase Inhibitor LXN Expression in Endometrial Tissue Is Menstrual Cycle Phase-Dependent and Is Upregulated in Endometriotic Lesions

**DOI:** 10.3390/genes15081086

**Published:** 2024-08-17

**Authors:** Meruert Sarsenova, Artjom Stepanjuk, Merli Saare, Sergo Kasvandik, Pille Soplepmann, Iveta Mikeltadze, Martin Götte, Andres Salumets, Maire Peters

**Affiliations:** 1 Department of Obstetrics and Gynaecology, Institute of Clinical Medicine, University of Tartu, 50406 Tartu, Estonia; meruert.sarsenova@ut.ee (M.S.); merli.saare@ut.ee (M.S.); andres.salumets@ki.se (A.S.); 2 Department of Women’s and Children’s Health, Division of Obstetrics and Gynecology, Karolinska Institutet, and Karolinska University Hospital, 17177 Stockholm, Sweden; 3 Competence Centre on Health Technologies, 50411 Tartu, Estonia; tjomas91@gmail.com (A.S.);; 4 Tartu University Hospital Women’s Clinic, 50406 Tartu, Estonia; pille.soplepmann@kliinikum.ee; 5 Department of Surgical and Gynecological Oncology, Tartu University Hospital, 50406 Tartu, Estonia; iveta.mikeltadze@kliinikum.ee; 6 Department of Gynecology, and Obstetrics, University Hospital of Münster, 48149 Münster, Germany; martin.goette@ukmuenster.de; 7 Division of Obstetrics and Gynaecology, Department of Clinical Science, Intervention and Technology (CLINTEC), Karolinska Institutet, and Karolinska University Hospital, 17177 Stockholm, Sweden

**Keywords:** *Latexin*, *LXN*, endometrium, endometriosis, gene expression, menstrual cycle

## Abstract

Endometriosis is a chronic hormone-dependent disease characterized by the spread of endometrial cells outside the uterus, which form endometriotic lesions and disrupt the functions of the affected organs. The etiopathogenesis of endometriosis is still unclear, and thus it is important to examine the genes that may contribute to the establishment of endometriotic lesions. The aim of this study was to investigate the expression of new potential candidate gene *latexin* (*LXN*), an inhibitor of carboxypeptidases, in endometrium and endometriotic lesions to elucidate its possible role in endometriosis development. *LXN* expression in tissues was assessed using quantitative reverse transcription PCR (qRT–PCR) analysis and immunohistochemical staining (IHC). The functions of *LXN* were examined using Transwell and MTT assays. qRT–PCR analysis revealed that *LXN* expression in endometrium was menstrual cycle-dependent, being lowest in the early-secretory phase and highest in the late-secretory phase and was significantly upregulated in endometriotic lesions. IHC confirmed LXN expression in endometrial stromal cells, and in vitro assays demonstrated that knockdown of LXN effectively reduced the migratory capacity of endometrial stromal cells while promoting cell viability. In conclusion, our results showed that LXN can be involved in the pathogenesis of endometriosis by regulating the proliferation and migration activity of endometriotic stromal cells.

## 1. Introduction

Endometriosis is a hormone-dependent gynecological disease that affects every tenth women of reproductive age. Endometriosis is commonly associated with clinical manifestations, such as chronic pelvic pain, menorrhagia and subfertility, and is characterized by the presence of endometrial-like glandular epithelium and stroma in the extrauterine locations that form lesions and affect the structure and function of the involved organs [1]. The etiopathogenesis of the disease is still under investigation and several factors, like inflammation, altered immune response, hormonal imbalance and interaction between endometrial cells and local microenvironment have been proposed to be critical for endometrial cell adhesion in ectopic locations [2,3]. Transcriptomic studies have identified that several genes involved in these cellular processes are differentially expressed in endometriotic lesions (ectopic endometrium) compared to eutopic endometrium (reviewed in [2]). Our previous transcriptomic analysis of fluorescent-activated cell sorted stromal cells from paired eutopic and ectopic endometrium revealed several differentially expressed genes [4], either already described as potentially involved in endometriosis pathogenesis or identified as novel in association with this condition. One of the novel genes, more abundantly expressed in ectopic stromal cells, was *latexin* (*LXN*), an inhibitor of carboxypeptidases [5]. An elevated level of LXN in peritoneal lesions was also noticed in our proteomics study of *in vitro* cultured stromal cells [6].

LXN is expressed at a moderate level across many human organs and tissues, including endometrium [7]. In malignant tissues, the expression of LXN is generally reduced compared to normal tissues, and based on molecular evidence that LXN possesses an inhibitory effect on cancer cell growth and tumorigenicity, a possible tumor suppressor function has been proposed [8,9]. Although loss of LXN in cells is thought to be involved in malignant processes, less is known about its function in non-cancerous tissues. There is evidence that LXN is a regulator for morphological maintenance of endothelial cells [10], plays an important role in the homeostatic hematopoiesis [11] and is a preadipocyte marker [12]. In mice, LXN is expressed at high levels in macrophages, and is further up-regulated in response to inflammatory stimuli [13], suggesting it plays role in inflammation. Interestingly, LXN level was found to be elevated in blood plasma of coronary artery disease patients, indicating plasma LXN as a potential biomarker of cardiovascular disease [14]. However, there is no data on the dynamics of *LXN* expression in endometrium or its possible role in endometriosis.

In this study, we examined the expression pattern of *LXN* throughout the menstrual cycle in the endometrium of women with and without endometriosis and explored its possible roles in the pathogenesis of endometriosis using *LXN*-silenced endometrial stromal cells. The results showed that *LXN* expression depends on the menstrual cycle and may play a role in cell migration and proliferation.

## 2. Materials and Methods

### 2.1. Study Participants

The study was approved by the Research Ethics Committee of the University of Tartu (approvals 276/M-13, 221/M-31 and 333/T-6). Written informed consent was obtained from all participants. Patients undergoing laparoscopic surgery because of endometriosis-specific symptoms (pelvic pain, infertility) were enrolled in the study from the Tartu University Hospital’s (TUH) Women’s Clinic (Tartu, Estonia). Women with endometriosis formed an ENDO group and patients who were not diagnosed with endometriosis during laparoscopy formed a non-endometriosis control group (non-ENDO). None of the participants had received hormonal treatment for at least three months prior to the time of sample collection. Tissue samples were collected, processed and preserved as described previously [4]. Endometrial samples were collected from 34 ENDO and 27 non-ENDO patients, in proliferative (P, n = 7 ENDO and n = 5 non-ENDO), early-secretory (ES, n = 8 ENDO and n = 13 non-ENDO), mid-secretory (MS, n = 10 ENDO and n = 7 non-ENDO) or late-secretory (LS, n = 9 ENDO and n = 2 non-ENDO) menstrual cycle phase [15]. Paired samples of eutopic and ectopic endometrium (endometriomas) were collected from 12 endometriosis patients (six in both P and MS phases). Nine paired eutopic and ectopic endometrium specimens were collected for immunohistochemical (IHC) analysis. The healthy group consisted of 12 volunteers with proven fertility who provided endometrial tissue samples during the ES and MS phase of the same menstrual cycle as described by Rekker et al. [16].

### 2.2. mRNA Extraction and Gene Expression Analysis

Total RNA from tissues was isolated using RNeasy Mini kit (Qiagen, Hilden, Germany) and cDNA was synthesized by RevertAid First Strand cDNA Synthesis Kit (Thermo Scientific, Waltham, MA, USA). mRNA was isolated from the cultured cells using the innuPREP RNA Mini Kit (Analytik Jena AG, Jena, Germany) following the manufacturer’s instructions and 1 µg of RNA was converted into cDNA using the High-Capacity cDNA Reverse Transcription Kit (Applied Biosystems, Darmstadt, Germany). The qRT-PCR analysis of *LXN* expression was performed using 200 ng of cDNA, 2× SYBR Select Master Mix (Applied Biosystems) or 5× HOT FIREPol EvaGreen qPCR Mix Plus (ROX) (Solis BioDyne, Tartu, Estonia) with the primers (F: 5′-GGTGTTTGAGGTGCAGAAGG-3′; R: 5′-TGCAGTTTCTTGTCCCGTTG-3′). Gene expression data was normalized to the *SDHA* housekeeping gene (F: 5′-TGGGAACAAGAGGGCATCTG-3′; R: 5′-CCACCACTGCATCAAATTCATG-3′). The 2-ΔΔCt method [17] was used for calculating the relative expression and to determine mRNA expression fold changes. The ΔCt values were calculated as follows: reference gene (*SDHA*) Ct value − Ct value of target gene (*LXN*).

### 2.3. Immunohistochemistry (IHC)

IHC analysis of tissue samples was performed to localize LXN protein in endometrium and endometriotic lesions. The tissue specimens were fixed in 10% neutral buffered formalin, dehydrated and embedded in paraffin at TUH’s Pathology Service. For IHC, 4 µm sections from paraffin-embedded tissue blocks were mounted on Superfrost Plus (Thermo Scientific, USA) slides. Tissue sections on slides were deparaffinized and rehydrated according to the standard protocol (Abcam IHC guide). Subsequently, for antigen retrieval, slides were heated in the water bath at 100 °C for 20 min in 10 mM Na-citrate buffer (pH 6.0) with 0.05% (*v*/*v*) Tween 20 (Naxo, Tartu, Estonia), then were cooled to room temperature and washed with tap water for 10 min. All following washing steps were done using 1× Tris-buffered saline (TBS) with 0.025% (*v*/*v*) Triton X-100 (PanReac AppliChem, Darmstadt, Germany) as a washing buffer. For staining, a mouse and a rabbit-specific HRP/DAB detection kit (ab64264, Abcam, Cambridge, UK) was used. All steps were performed according to the manufacturer’s protocol with a few modifications. After protein blocking and washing steps, additional blocking against possible tissue endogenous biotin was applied using the endogenous avidin-biotin blocking kit (ab64212, Abcam, UK). Mouse monoclonal antibody (clone OTI1E10) at a concentration 3.6 µg/mL, was used as a primary anti-Latexin antibody (MA5-25742, ThermoFisher Scientific, USA). Mouse IgG1 (MAB002, Biotechne R&D Systems, Minneapolis, MN, USA) was used for isotype control at the same concentrations as the primary antibody. Tissue section with primary antibody and mouse non-specific IgG subclasses were incubated overnight (16 h) in a humidity chamber at 4 °C. For antibody dilution and incubation, 1× TBS buffer with 1% (*w*/*v*) BSA (Capricorn Scientific, Ebsdorfergrund, Germany) was used. After incubation with the primary antibody, we performed an additional blocking step with 4% (*v*/*v*) normal goat serum (Abcam, UK) diluted in 1× TBS buffer. Further steps were performed as per the manufacturer’s protocol. The chromogenic reaction was developed for 30 s and stopped after that. Cell nuclei were counterstained with Mayer’s haematoxylin solution. Slides were examined under an Olympus BX41, and tissue microphotos were taken with an Olympus DP71 camera and CellˆB (Olympus, Tokyo, Japan) software.

### 2.4. Cell Culture and siRNA Transfection

The immortalized human endometrial stromal cell line St-T1b [18] was used for *LXN* siRNA transfection. St-T1b was cultured in medium containing 70% Dulbecco’s modified Eagle’s medium (DMEM) (PAA by GE Healthcare Life Sciences, Chalfont St Giles, UK), 18% MCDB-105, supplemented with 10% fetal calf serum, 1% glutamine, and penicillin/streptomycin/insulin (100 U/mL, 100 μg/mL, and 5 μg/mL, respectively). For siRNA transfection, cells were plated in six-well plates 1 day before transfection to reach 70% confluency. Cells were then transfected with *LXN* siRNA (Cat 4392420, Ambion by Thermo Fisher Scientific, USA) or negative control siRNA #1 (cat 4390844, Ambion, USA), via lipotransfection with DharmaFECT reagent (Thermo Fisher Scientific, USA) in OPTI-MEM media (Life Technologies, Grand Island, NY, USA) according to the manufacturer’s instructions. After 24 h, the transfection medium was replaced with the culture medium normally used for St-T1b culturing. *LXN* mRNA expression in *LXN* siRNA transfected cells was measured by qRT-PCR 48 h after transfection and showed 86–90% decrease in *LXN* expression.

### 2.5. MTT (3-(4,5-Dimethyl-2-yl)-2,5-Diphenyltetrazolium Bromide) Cell Viability Assay

Forty-eight hours after siRNA transfection, 5000 of St-T1b cells were seeded in 96-well plates and cultured for 24 h, followed by a 24-h incubation in the presence of methylthiazolyldiphenyltetrazolium bromide (MTT). Further, the cells were lysed and the optical density measurement at 595 nm was performed in a microplate reader. The proliferation of the control cells was defined as 100%.

### 2.6. Migration Assay

To assess whether the suppression of *LXN* expression affects cell migration ability, 8.0 µm pore size transwell chambers (Falcon) were placed in a 24-wells plate containing 600 µL of serum-free migration medium. A 100 µL of cell suspension (15,000 cells) was added at the top compartment, and the cells were allowed to migrate for 36 h. After migration, transwells were washed once with PBS, fixed in methanol, stained with DiffQuik (Medion Diagnostics, Düdingen, Switzerland) and photographed utilizing a Zeiss Axiovert microscope equipped with Axiovision software (Zeiss, Jena, Germany) at 50× magnification. The cells in the two central visual fields of every membrane were counted for analysis of migratory activity.

### 2.7. Statistical Analysis

The data are presented as the mean ± SD from at least three independent experiments. All statistical analyses were performed using GraphPad Prism 10 (San Diego, CA, USA) software. Statistical significance was measured using one-way ANOVA or Student’s *t*-test. In all figures, differences were considered significant at *p* < 0.05, and denoted as * for *p* < 0.05, ** for *p* < 0.01, *** for *p* < 0.001, and **** for *p* < 0.0001.

## 3. Results

### 3.1. Characteristics of the Study Population

The general characteristics of the study participants are given in Table 1. The ages and BMIs of the women in the different groups (ENDO, non-ENDO and healthy) were not statistically significantly different (unpaired *t*-test was applied). The differences were also not statistically significant in the comparisons where the study groups were divided according to the phases of the menstrual cycle (one-way ANOVA, *p*-values 0.96 and 0.79 for age and BMI, respectively).

### 3.2. LXN Expression in Eutopic and Ectopic Endometrium

**Figure 1:**
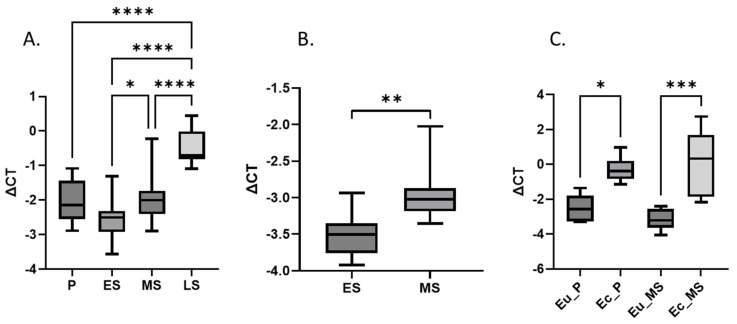
*LXN* mRNA expression in endometrium and endometriotic lesions. (**A**) Expression of *LXN* in endometrium throughout the menstrual cycle (in a combined group of ENDO, n = 34 and non-ENDO, n = 27). (**B**) Expression of *LXN* in endometrium of healthy women in ES (n = 12) and MS phases (n = 12). (**C**) Expression of *LXN* in paired eutopic and ectopic endometrium of endometriosis patients (n = 12). Statistical difference is shown only for comparisons where it is significant. P: proliferative phase, ES: early-secretory phase, MS: mid-secretory phase, LS: late-secretory phase, Eu: eutopic endometrium, Ec: ectopic endometrium. *Y*-axis shows relative *LXN* mRNA level normalized by *SDHA* (ΔCT). One-way ANOVA was applied, **** correspond to *p* value < 0.0001, *** *p* value < 0.001, ** *p* value < 0.01, * *p* value < 0.05.

To detect whether the expression of *LXN* in eutopic endometrium is affected by the menstrual cycle phase, we analyzed *LXN* mRNA levels by qRT-PCR in a total of 61 endometrial samples (Table 1) collected throughout the menstrual cycle from 34 and 27 ENDO and non-ENDO group women, respectively. The analysis did not reveal statistically significant differences between the samples from both groups collected in the same menstrual cycle phase (all *p* > 0.05, LS phase samples were not compared as there were only two LS samples in non-ENDO group). Therefore, the samples from both study groups were combined and 12, 21, 17 and 11 endometrial samples from P, ES, MS and LS menstrual cycle phases, respectively, were used for the cycle phase-specific *LXN* expression analysis. The results demonstrated that *LXN* expression level in eutopic endometrium was significantly different between menstrual cycle phases (one-way ANOVA *p* < 0.0001), with the lowest expression in ES and the highest expression in LS phase (Figure 1A).

Next, we analyzed *LXN* expression from endometrial tissues collected from 12 healthy volunteers in hormonally confirmed [according to luteinizing hormone (LH) level] ES (pre-receptive) and MS (receptive) phases. Similar to the samples from ENDO group, the expression of *LXN* increased 1.5-fold in MS compared to ES phase endometrium (Student’s paired *t*-test *p* = 0.002, Figure 1B). Interestingly, we observed moderate but statistically significantly higher expression of *LXN* both in both ES and MS samples of patients compared to healthy women (2.0-fold and 1.9-fold, respectively, one-way ANOVA both *p* < 0.0001).

Furthermore, the expression of *LXN* was assessed in 12 paired endometrial and ovarian endometrioma tissues (6 samples both from P and MS phases). The results demonstrated on average 4.6-fold and 9.8-fold higher *LXN* expression in P and MS phase endometriomas compared to eutopic endometrium, respectively (one-way ANOVA, *p* = 0.01 and *p* = 0.0003, respectively) (Figure 1C).

### 3.3. LXN Expression in Endometrial Tissue by IHC

**Figure 2:**
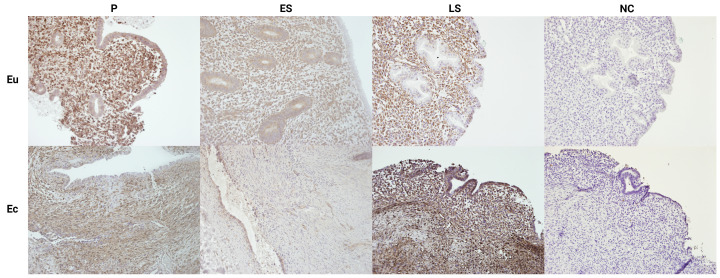
Representative IHC images of LXN expression in eutopic (Eu) and ectopic (Ec) endometrium in different menstrual cycle phases. P: proliferative phase, ES: early-secretory phase, LS: late-secretory phase, NC: negative control. Original magnification ×200.

IHC analysis was performed on 9 endometrial and 6 endometriotic lesion samples from P, ES and LS menstrual cycle phases. The analysis showed that LXN protein in endometrium and endometriotic lesions was mostly localized in the stromal compartment (Figure 2), and weaker expression was detected in the glandular epithelial cells. Overall, the LXN protein expression in tissues mirrored what we observed at mRNA level—the expression was higher in P and LS phase, compared to ES phase, and higher in lesions compared to eutopic endometrium. However, as only limited number of tissue sections was available from endometriotic lesions (e.g., only one lesion from ES phase, Table 1) we did not evaluate the statistical significance of expression differences based on IHC staining.

### 3.4. LXN Silencing Affects Endometrial Stromal Cell Migration and Viability

**Figure 3:**
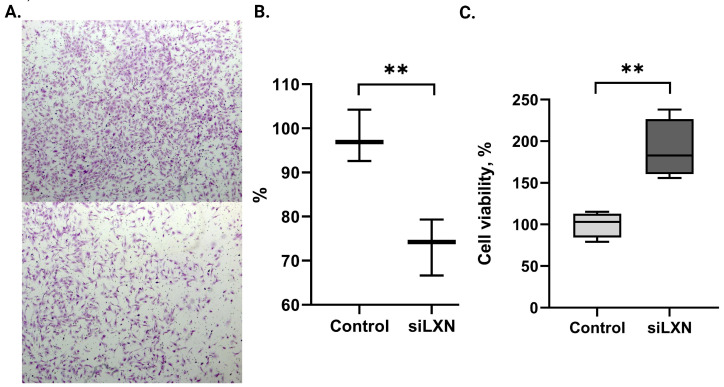
Silencing of *LXN* affects the migration and viability of endometrial stromal cells. (**A**) Migration capability of St-T1b cells treated with control (upper panel) or *LXN*-siRNA (siLXN, lower panel) was assessed by Transwell assay and the migrated cells were quantified (original magnification ×50). (**B**) The migration ability of siLXN treated cells is calculated as a percentage of control mean. (**C**) The viability of St-T1b cells after transfection with *LXN*-siRNA and control siRNA. The viability of the control cells was defined as 100%. ** Correspond to *p* value < 0.01.

To investigate the role of LXN in endometrial stromal cells, we performed functional experiments using the immortalized endometrial stromal cell-line St-T1b. As *LXN* is naturally highly expressed in stromal cells, we transfected the cells with *LXN*-siRNA to suppress the *LXN* gene expression and determined whether reducing its level affects cell migration and viability, i.e., functions that are important for endometriosis pathophysiology. The results revealed that suppression of *LXN* in St-T1b cells transfected with *LXN*-siRNA resulted in reduction of migration to approximately 72% compared to cells transfected with control siRNA (*p* = 0.008; Figure 3A,B).

MTT assay was performed to assess the effect of *LXN* silencing on cell viability. In contrast to the effect on cell migration, *LXN*-siRNA transfected St-T1b cells showed higher viability compared to control siRNA transfected cells (1.7-fold increase, *p* = 0.003) (Figure 3C).

### 3.5. LXN Availability in Body Fluids

We have previously analyzed the protein composition of uterine fluid collected from fertile women in the ES and MS phases using liquid chromatography tandem-mass spectrometry (LC-MS/MS) [19]. According to previously published data, LXN may be a secreted protein [20]; therefore, we examined the available LC-MS/MS data for the presence of LXN in the uterine fluid. We discovered that while LXN was not statistically differentially expressed between the samples from different menstrual cycle phases, it was one of over 3000 proteins consistently detected in all analyzed uterine fluid samples (data accessible from Table S4a in [19]), suggesting possible secretion from uterine cells.

We also re-examined our LC-MS/MS blood plasma proteome data obtained from the analysis of pooled ENDO and non-ENDO plasma samples (24 pools of 119 ENDO blood plasma samples and 12 pools of 53 non-ENDO blood plasma samples) [21] to investigate LXN presence in the circulation. Unfortunately, peptides corresponding to the LXN protein were detected in less than half of the samples: in the ENDO group, one peptide corresponding to LXN was detected in 5 and two peptides in 3 of 24 plasma pools. Of the 12 pools in the non-ENDO group, one and two peptides corresponding to LXN were found in 5 and 1 pools, respectively. () This result indicates that LXN either does not enter the bloodstream or cannot be determined from blood samples by the methodology used.

## 4. Discussion

Although the carboxypeptidase inhibitor LXN has been widely studied in cancer, there is still little knowledge about its expression and possible role in non-cancerous tissues. The results of our study have extended this knowledge to normal endometrium and endometriosis. We found that the expression of *LXN* in endometrial tissue is menstrual cycle-dependent and is increased in endometriotic lesions compared to eutopic endometrium. In vitro experiments suggested that LXN may be involved in cellular processes that are also important for the development of endometriotic lesions, such as endometrial stromal cell migration and viability.

The ovarian hormones influence the expression of many genes in endometrial tissue. Therefore, while investigating gene expression changes in endometrium-related diseases, such as endometriosis, the menstrual cycle impact on the particular gene expression level should be considered. However, there was no data about *LXN* expression neither in endometrium throughout the menstrual cycle nor in endometriosis. We found that the mRNA level of *LXN* was similar in the endometrial samples from women with and without endometriosis and changed throughout the menstrual cycle, being the highest in the LS phase. Increase of *LXN* level in MS compared to ES phase endometrium was also observed in tissue samples from healthy fertile females, and interestingly, the *LXN* expression was higher in both phases in samples collected from ENDO and non-ENDO patients compared to healthy women. However, this result has to be taken with caution as the difference is probably caused rather by the distinct characteristics of the samples than because of the presence of disease. ES and MS phase endometrial samples from the healthy group were collected from the same women during one menstrual cycle on days determined by the LH test (respectively 2 and 7 days after the LH peak), and the occurrence of ovulation was confirmed by ultrasound [16], thus reducing some individual-specific variability between samples. The cycle phases of endometrial samples from ENDO and non-ENDO patients were determined according to the self-reported cycle days and even though some corrections in ES and MS phase sample groups were made as described by Saare et al. [15], the groups were not as uniform. The fact that samples from healthy women were collected using local anesthesia and samples from ENDO group during laparoscopy may also contribute to this difference between the groups.

Further, we analyzed the *LXN* expression in paired endometrial and endometriotic tissue samples from proliferative and secretory phases separately. We observed higher *LXN* expression in proliferative phase endometrioma samples compared to eutopic endometrium, which confirms the results of the previous transcriptomics study on a pure population of stromal cells [4]. The difference was also evident between the eutopic and ectopic samples in the secretory phase, but in this phase, the *LXN* expression level varied more in endometriomas.

Since stromal cells in lesions possess higher *LXN* expression compared to eutopic cells, we can hypothesize that lesions are established either by a small subset of eutopic stromal cells with higher migratory properties and higher *LXN* level, or alternatively, *LXN* may be induced in cells of an ectopic location by compounds of the surrounding environment. For example, it has been suggested that macrophages and endometrial stromal cells communicate with each other as the factors secreted by macrophages alter the expression of several genes, including LXN, in human endometrial stromal cells and this communication may support the development of endometriosis [22]. Therefore, the high expression of *LXN* in endometriotic lesions may be induced by some macrophage-secreted inflammatory compounds in peritoneal environment.

The higher *LXN* expression in lesions raised the question of cellular functions of this protein in endometrial cells. A previous study showed that suppression of *LXN* by siRNA decreased the motility of prostate epithelial cells and ectopically overexpressed *LXN* restored the migratory activity of the cells [23]. Our data showed that suppression of *LXN* in St-T1b cells resulted in a decrease in a migratory activity, suggesting that LXN is involved in endometrial cells’ motility in a similar manner as in prostate cells. Ovarian steroids have a greater stimulatory effect on migration/invasion of cells derived from women with endometriosis compared to women without the disease [24], but the data on basal migratory ability of untreated endometrial cells from lesions and endometrium are contradictory. Ornek et al. showed that invasiveness of endometrial stromal cells from lesions is higher compared to stromal cells from the paired eutopic endometrium and endometrial stromal cells from women without endometriosis had the lowest invasive characteristics [25]. On the contrary, Yotova et al. demonstrated that migratory ability of ectopic stromal cells from endometriomas was considerably lower compared to eutopic cells [26]. The regulation of motility and invasion of endometrial stromal cells is a complex process regulated by many factors that may differentially affect eutopic and ectopic stromal cells [27]. In addition, the mobility of endometrial cells is affected by tissue maturation processes occurring during the menstrual cycle [28]. Thus, changes in genes implicated in cell motility may be associated with other important endometrial functions and may thereby contribute to endometriosis pathogenesis.

The proliferative activity of endometrial cells peaks in the preovulatory phase and shows no difference between women with and without endometriosis. However, the proliferative activity of endometriotic cells in lesions is notably lower compared to eutopic endometrial cells across the menstrual cycle [29]. We observed that silencing of *LXN* increased the proliferation/viability of stromal cells, indicating that higher *LXN* expression in endometriotic cells may have a negative effect on cell proliferation. This assumption is also supported by previous studies on *LXN* low-expressing cancer cell lines, where exogenous expression of *LXN* resulted in inhibition of cell growth [8,9].

Based on empirical evidence, it has been suggested that even without a signal peptide, LXN is secreted from prostate cells through either the release of extracellular vesicles or a non-exosomal pathway [20]. The presence of LXN in uterine fluid implies its potential secretion from uterine tissue. The total proteome of uterine fluid consists of extracellular, secreted, or plasma membrane-released proteins, with proteins of extracellular vesicles being notably enriched [19]. Hence, it is plausible to hypothesize that LXN may enter uterine fluid as a component of extracellular vesicles. Unfortunately, LXN was not so easily detectable in blood plasma in our study. Consequently, although LXN has been suggested as a blood-based cardiovascular disease biomarker [14], it may not be a feasible non-invasive biomarker candidate for endometriosis. Li et al. [14] recognized the absence of a comprehensive methodology for LXN determination in plasma and developed a unique specific electrochemical immunosensor system for this purpose. Thus, we cannot dismiss the possibility that LXN plasma levels are altered in endometriosis; however, the LC-MS/MS method we employed is unsuitable for detecting its presence in plasma.

Our study has some limitations. The first limitation is the small number of study participants; therefore, differences between some groups may have been missed. Due to this limitation, we were also unable to assess statistical significance in LXN expression using IHC results. Additionally, we did not quantify LXN protein levels using Western blot due to a lack of suitable tissue samples. Second, for *LXN* mRNA expression analysis in paired samples of endometrium and lesions, we only used endometrioma samples. Nevertheless, since our previous proteomics study [6] showed higher levels of LXN in peritoneal lesions stroma, it can be assumed that it correlates with mRNA expression in peritoneal lesions. Third, as mentioned above, the difference in *LXN* gene expression in endometrial samples from healthy controls compared to ENDO and non-ENDO group women could be influenced by sample collection characteristics.

## 5. Conclusions

Our study has expanded knowledge on the expression and possible functions of the relatively unexplored *LXN* gene in the context of endometrium and endometriosis. We showed that *LXN* is expressed in normal endometrial tissue in a menstrual cycle-dependent manner and to our knowledge, this study is the first to demonstrate that LXN may have a role in endometrial cell proliferation and migration. *LXN* expression is elevated in endometriotic lesions; however, it is unclear whether the high *LXN* level plays a crucial role in the development of endometriosis or if its expression is increased due to the local effects from peritoneal environment following the formation of endometriotic lesions.

## Figures and Tables

**Table 1:** General characteristics of the study participants.

	Gene Expression Analysis of Endometrial Tissues throughout the Menstrual Cycle			mRNA and Protein Expression Analyses of Paired Samples of Endometrium and Lesions	
	ENDO (n = 34) ^a,b^	Non-ENDO (n = 27) ^a,c^	Healthy (n = 12)	Gene Expression (n = 12)	IHC (n = 9 ^d^)
Age (years, SD) *p*-value *p*-value	31.2 ± 4.5 0.48 ^a^ 0.58 ^b^	32.1 ± 5.6 0.48 ^a^ 0.34 ^c^	30.4 ± 3.7	31.6 ± 4.9	34.3 ± 6.5
BMI (kg/m^2^, SD) *p*-value *p*-value	22.3 ± 3.5 0.29 ^a^ 0.62 ^b^	23.2 ± 3.7 0.29 ^a^ 0.80 ^c^	22.9 ± 4.6	23.1 ± 3.5	22.7 ± 3.7
Infertility ^e^ (n, %)	24 (70.6%)	20 (75.1%)	NA	6 (50%)	4 (44%)
N of children, SD	0.4 ± 0.7	0.5 ± 0.8	1.3 ± 0.5	0.7 ± 1.1	0.9 ± 1.4
Endometriosis stage (%)	I–II (53%) III–IV (47%)	NA	NA	III–IV (100%)	I–II (56%) III–IV (44%)
Menstrual cycle phase (n)	P (7), ES (8), MS (10), LS (9)	P (5), ES (13), MS (7), LS (2)	ES (12), MS (12)	P (6), MS (6)	P (3), ES (3), LS (3)

^a^ *p*-value for the comparison ENDO vs. non-ENDO; ^b^ *p*-value for the comparison ENDO vs. healthy; ^c^ *p*-value for the comparison non-ENDO vs. healthy; ^d^ the number of endometrial biopsies was 9 and endometriotic lesions was 5 (2 in P, 1 in ES and 2 in LS phase); ^e^ infertility at the moment of participating in the study; ENDO: women with endometriosis, non-ENDO: women without endometriosis, IHC: immunohistochemistry; P: proliferative phase; ES: early-secretory phase; MS: mid-secretory phase; LS: late-secretory phase; NA: not applicable; SD: standard deviation.

## Data Availability

The original contributions presented in the study are included in the article, further inquiries can be directed to the corresponding author/s.
